# Assessing cycling-friendly environments for children: are micro-environmental factors equally important across different street settings?

**DOI:** 10.1186/s12966-015-0216-2

**Published:** 2015-05-02

**Authors:** Ariane Ghekiere, Jelle Van Cauwenberg, Lieze Mertens, Peter Clarys, Bas de Geus, Greet Cardon, Jack Nasar, Jo Salmon, Ilse De Bourdeaudhuij, Benedicte Deforche

**Affiliations:** Department of Public Health, Faculty of Medicine and Health Sciences, Ghent University, De Pintelaan 185, 4K3, B-9000 Ghent, Belgium; Department of Human Biometry and Biomechanics, Faculty of Physical Education and Physical Therapy, Vrije Universiteit Brussel, Pleinlaan 2, B-1050 Brussels, Belgium; Fund for Scientific Research Flanders (FWO), Egmontstraat 5, B-1000 Brussels, Belgium; Department of Movement and Sport Sciences, Faculty of Medicine and Health Sciences, Ghent University, Watersportlaan 2, B-9000 Ghent, Belgium; Department of Human Physiology, Faculty of Physical Education and Physical Therapy, Vrije Universiteit Brussel, Pleinlaan 2, B-1050 Brussels, Belgium; Ohio State University, City and Regional Planning, 292 Knowlton Hall, West 18 Woodruff Avenue 275, Columbus, OH 43210 USA; Centre for Physical Activity and Nutrition Research, School of Exercise and Nutrition Science, Deakin University, Melbourne, Australia

**Keywords:** Built environment, Road characteristics, Bicycle, Preference, Youth, Simulated environments, Experiment

## Abstract

**Background:**

As physical activity levels decrease as children age, sustainable and accessible forms of physical activity are needed from a young age. Transportation cycling is one such physical activity and has been associated with many benefits. The aims of the study were to identify whether manipulating micro-environmental factors (e.g. speed limits, evenness of cycle path) within a photographed street influences the perceived supportiveness for transportation cycling; and whether changing these micro-environmental factors has the same effect across different street settings.

**Methods:**

We recruited 305 fifth and sixth grade children and their parents from twelve randomly selected primary schools in Flanders, Belgium. They completed a web-based questionnaire including 12 choice-based conjoint tasks, in which they had to choose between two possible routes depicted on manipulated photographs, which the child would cycle along. The routes differed in four attributes: general street setting (enclosed, half open, open), evenness of cycle path (very uneven, moderately uneven, even), speed limit (70 km/h, 50 km/h, 30 km/h) and degree of separation between a cycle path and motorised traffic (no separation, curb, hedge). Hierarchical Bayes analyses revealed the relative importance of each micro-environmental attribute across the three street settings.

**Results:**

For each attribute, children and their parents chose routes that had the best alternative (i.e. open street setting, even cycle path, 30 km/h, a hedge separating the cycle path from motorised traffic). The evenness of the cycle path and lower speed limit had the largest effect for the children, while the degree of separation and lower speed limit had the largest effect for their parents. Interactions between micro-scale and macro-scale factors revealed differences in the magnitude but not direction of their effects on route choice. The results held across the different kinds of street settings tested.

**Conclusions:**

Improving micro-scale attributes may increase the supportiveness of a street for children’s transportation cycling. We call for on-site research to test effects of changes in micro-environmental attributes on transportation cycling among children.

**Electronic supplementary material:**

The online version of this article (doi:10.1186/s12966-015-0216-2) contains supplementary material, which is available to authorized users.

## Background

Most children (i.e., around 65 percent) do not achieve the recommended hour of physical activity (PA) a day [1]. As children age, their PA levels decline, making them an important group to focus on in PA promotion [2]. Cycling for transport is an inexpensive type of PA which children can integrate into their daily routine in most geographical regions [3]. Children who regularly cycle for transport are physically and mentally healthier than their non-cycling peers [4,5]. Before children reach the age of ten, their parents tend to control their outdoor movements from place to place [6,7], after which the parents give them more leeway and the children take a more active role in their active transport related decisions [8,9]. Thus, it makes sense to consider both the parents’ and children’s perspectives when studying influences on transport behaviour.

The decrease in PA levels during the transition from childhood to adolescence and the increase of independent mobility from around ten years of age make children from the upper years of primary school an important target group for the promotion of cycling for transport. Although researchers have observed some differences in cycling rates between European countries [10], cycling rates are generally low. In Flanders (Belgium), only 11% of children aged 6–12 years old use cycling as their main transport mode [11]. To develop interventions aiming to increase cycling for transport among this specific age group, an understanding of the key determinants is needed [12].

As suggested by socio-ecological models, possible determinants of cycling for transport among children include individual (e.g. attitudes and beliefs), and physical (e.g. specific route characteristics) and social (e.g. family) environmental factors [13]. However, these variables affect an individual’s choice to cycle at different time points of the decision-making process, implying that some factors may be more important than others [14]. Alfonzo suggests that walking (and other forms of physical activity such as cycling) is influenced by a hierarchy of factors [15], which implies that a more basic need has to be satisfied before needs of a higher order come into play. Studies of the association between children’s cycling for transport and the physical environment showed consistent associations with macro-environmental elements. For example, longer distances to destination showed negative associations with cycling for transport [16], while elements from the ‘walkability index’ (i.e., street connectivity, residential density and mixed-land use [17]) were positively associated with the share of transportation cycling [18,19]. Although these elements fit into the two basic layers in the hierarchy, i.e. feasibility and accessibility [15], they are difficult to modify in existing neighbourhoods. Micro-scale environmental factors, which fit into the three upper layers of the hierarchy: safety, comfort and pleasurability [15], are easier to modify. However, the associations of micro-environmental factors with children’s transportation cycling require additional study. Physical characteristics of cycling infrastructure such as width of the cycle lane, evenness of the surface, degree of separation with the motorised traffic and cars’ speed limits show inconsistent associations with levels of cycling for transport among children [9,20,21]. These inconsistencies may result from measurement issues [22].

Previous studies often used self-report questionnaires to assess neighbourhood physical environmental characteristics. However, such questionnaires have at least three limitations. First, they require the participant to recall specific, detailed environmental factors while not in that environment, and this may result in a mismatch with the actual neighbourhood characteristics [23]. Second, when asked to describe physical elements within their neighbourhood, participants do not receive a proper definition of the “neighbourhood” [24,25]; and as a result, they may answer with a different neighbourhood in mind than that intended by the researchers. Third, physical characteristics of a neighbourhood may co-vary, such that the cause of an association with cycling would remain uncertain.

The use of photographs can overcome these problems in that they neither require participants to recall an environment nor respond based on their impression of their neighbourhood [19-22]. They also allow one to create controlled manipulations of physical micro-environmental factors (e.g. evenness of cycle path, speed limits etc.) to examine the causal relationship between each environmental factor and the participant’s response, which otherwise only more expensive and time-consuming longitudinal, natural experiments can find.

Two recent studies in adults used manipulated photographs to examine which factors in the environment influenced the appeal of walking [26] or cycling [27]. The studies manipulated panoramic photos on several micro-environmental factors (e.g. even vs. uneven cycle path, the presence vs. absence of vegetation etc.). Both studies succeeded in identifying the effect of changing physical micro-environmental factors on the appeal of a street environment to walk or cycle along. However, because these studies centered on one type of streets (a typical urban street in Flanders), the generality of their findings to other street settings remains uncertain. Current research has not yet identified whether particular micro-environmental factors in a street relate to its appeal for transportation cycling in children; nor has it found out whether changing micro-environmental factors can improve the perceived supportiveness among children for transportation cycling across different street settings. At the same time, we need more insight in the generalizability of findings among adults [26,27], since the effect of an environmental factor may vary with the type of street. For example, a separated cycle path may be more important in urban, densely built streets than in a less-urban lower density street. It seems likely that higher preferences for lower motorised traffic speed would occur for streets with a high land-use mix, as such streets may have more road users, making cycling more difficult and less comfortable.

The present study had two purposes: first, it sought to identify whether manipulating micro-environmental factors within a street influences fifth and sixth grade children’s and their parents’ perceptions of a street’s appeal for cycling for transport; and second, it sought to identify whether changing these micro-environmental factors has the same effect across different street settings. To answer both research questions, the current study used manipulated photographs, integrated in choice-based conjoint tasks. Based on previous findings [9,18,21,28,29], we hypothesized that children and their parents would prefer the anticipated best level within each environmental factor. Furthermore, for the children, we hypothesized that the evenness of the cycle path would be the most important factor, because children might be more aware of its hampering their ability to cycle [30,31], but for their parents we hypothesized that motorised traffic speed and degree of separation would be more important because of the parents’ concern for traffic safety [9,18,21,28,29].

## Methods

### Recruitment and procedure

Twenty randomly chosen schools located across Flanders (Belgium) were contacted by telephone and asked to participate in the study. Twelve schools agreed to participate (response rate = 60%). All schools were visited twice: during the first visit, a researcher explained the aim of the study to all pupils from 5^th^ and 6^th^ grade in each primary school (n = 703). Children received an invitation letter for their parents to participate in the study. In this letter, parents were given information about the study and asked to give written consent for their child to participate and complete an online questionnaire at school. For parents who were prepared to participate themselves, a personalised login and password was included in the letter to complete an online parental questionnaire at home. After one week, the researchers returned to the schools to collect consent forms and to assist children in completing the online questionnaire. All data were collected between March and April 2014. The study protocol was approved by the Ethics Committee of the University Hospital of Brussels.

### Development of the photographs

**Figure 1:**
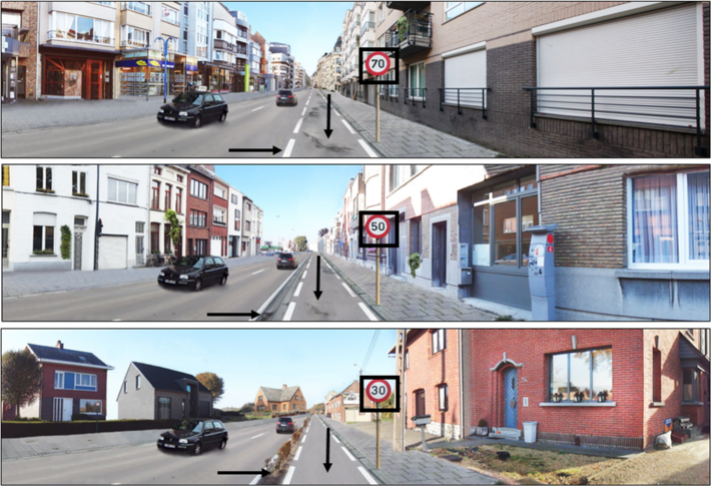
The four environmental factors of interest included in the current study, with their respective levels: 1. General street setting; 2. Evenness of cycle path; 3. Speed limit; 4. Degree of separation.

Prior to data collection, panoramic colour photographs were developed with Adobe Photoshop© software, depicting a possible route to cycle along. Previous research showed good validity of responses to colour photographs compared to on-site responses [32]. In this study, we opted to first examine whether a set of three micro-scale environmental factors are equally important across different street settings. As it would be impractical to include all micro-scale environmental factors within the different street settings, this step should represent an intermediate step before including all potential micro-scale environmental factors. Therefore, we manipulated the panoramic photographs on four attributes: the general street setting (enclosed, half-open, open) and three micro-environmental attributes (evenness of cycle path, degree of separation from motorised traffic, and the speed limit for motorised traffic) (see Figure 1). A previous study had highlighted the importance of these four environmental factors for cycling for transport in children [18]. The photographs were developed from a cyclist’s view-point in order to obtain a street view which is similar to that experienced during cycling.

Each environmental attribute consisted of three levels, including an unattractive level, an intermediate level and an attractive level (resulting in 81 photographs being created, 3 levels by 4 attributes). The environmental attributes consisted of the following levels: *evenness of the cycle path*, i.e., very uneven, moderately uneven, even; *speed limit*, i.e., 70 km/h, 50 km/h, 30 km/h; and *degree of separation from motorised traffic*, i.e., no separation, a curb, a hedge. The street settings were established aiming to generate three different street views. The first street setting was created to get an enclosed street view, with high residential density and a mixed land-use including residences, shops and banks. The second street setting was created to obtain a typical (semi-)urban street in Flanders, with a high residential density and a single land-use (residences only). A third street setting was created with a low residential density and single land-use, creating a more open street setting. A number of elements were kept constant across all photographs to increase standardisation of the protocol: all photographs were created with two cars on the road, a wide cycle path, good weather conditions and no other people on the foot path or cyclists on the cycle path (Figure 1).

### Study protocol

Children and their parents completed a web-based questionnaire constructed with Sawtooth SSI Web® (v8.2.4).

#### Children

In the first part of the questionnaire, children reported their height, weight, sex, date of birth, school, current grade (5^th^ or 6^th^ grade), place of residence and their usual transport mode to school. Children’s Body Mass Index (BMI) was calculated as their weight divided by their squared height in meters (kg/m^2^). Furthermore, children were asked to indicate who generally decides their transport mode to school (response options: mother, father, both parents, both parents and the child, the child on his/her own) and who accompanies them to school (response options: nobody, parents, friends, brothers/sisters, parents with brother/sister/friends).

In the second part of the questionnaire, children completed a choice-based conjoint (CBC) task. Conjoint analysis is a quantitative market research technique that asks participants to choose among multiple ‘products’, in this case ‘streets’. Each product was described using the levels (e.g., very uneven, 30 km/h, and no separation) of the relevant attributes (e.g. evenness of the cycle path, speed limit, and degree of separation from motorised traffic) [33] and participants were asked to indicate which street they preferred most. Each question in CBC is usually called a choice task, with most studies using a set of 12 to 15 choice tasks per participant [33]. CBC analysis benefits from the fact that participants do not have to choose between all possible combinations of attributes (environmental factors) but the software assigns choice combinations randomly to each participant. Most CBC studies used written descriptions of the attribute levels of the products, but photographs have also been used successfully in research settings [34].

Children received the following scenario: “Imagine that you are going to visit your friend who lives 10 minutes by bike from your residence. It’s a beautiful day, not too hot, not too cold and dry. Which route would you prefer to cycle to your friend? You will get to see two streets. The aim is that you choose the street that you would prefer to cycle to your friend. The time to cycle to your friend is the same along the two routes. Take sufficient time to inspect the photographs.” This instruction ensures standardisation for distance, as it is known to be a key determinant for active transport among children [16], but also for weather conditions [35] and destination of the cycle trip. Children had to choose one of the two provided alternatives to cycle along, which is similar to an everyday situation in which the cyclist needs to choose between available routes. A full profile design was used, meaning that all four environmental attributes could be manipulated in each pair of photographs presented. Prior to the actual choice-tasks, children were shown three examples of CBC tasks to get familiar with the manipulated photographs used in the tasks. After finishing these three trials, children completed 12 randomly assigned choice tasks.

#### Parents

Parents first completed questions about their level of education (less than secondary school; secondary school; college or university degree), marital status, and walking and cycling behaviour in a usual week, and questions about their child (height, weight, walking and cycling behaviour in a usual week [36]). Then, they chose which route they would prefer their child to cycle independently (i.e. without supervision of an adult) to visit a friend living 10 minutes from their residence. Except for this difference in instruction, the parental CBC task was identical to the task among children.

### Analyses

Descriptive characteristics of the children and their parents were computed in SPSS version 22. The analyses were conducted on the sample of children for whom parental data were available, so that we could draw inferences from the sample of children and their corresponding parents. Differences between the included and excluded children were examined with an independent samples *t*-test for continuous variables and chi^2^-tests for categorical variables. All other analyses were performed for parents and children separately. Power calculations were performed before data collection to establish the appropriate number of participants via a CBC rule of thumb; $\frac{nta}{c}>500$, with *n* = *number of participants*, *t* = *number of tasks*, *a* = *number of alternatives per tasks* and *c* = *number of analysis cells* [33]. When considering all two-way interactions, c is equal to the largest product of levels of any two attributes [37]. In the current study, 12 tasks were developed with two alternatives per task and the largest product of the levels of any two attributes was 9 (all attributes/environmental factors had 3 levels). Therefore, a sample size of 188 participants was sufficient for the current study design.

Individual preferences for all environmental attribute levels, called part-worth utility scores, were estimated with Hierarchical Bayes analysis in Sawtooth Software®. Hierarchical Bayes analysis is considered as the best method to CBC data [33,38]. This analysis assumes heterogeneous preferences and allows estimation of the mean (average part-worth utilities) and variance of level preference weights for each participant [38]. These part-worth utilities can be considered as the preference for an attribute level, whereas total utilities can be considered as the total desirability of an environmental attribute in the CBC tasks. Greater positive values of a part-worth utility indicate a higher preference for that specific level within the environmental attribute [33]. Individual part-worth utilities are used to calculate individual total utilities, which are averaged for the total sample to describe the results.

Four separate models were estimated; one including all main effects of the four environmental attributes and three separate models adding each time the interaction effect between street setting and one micro-environmental attribute. For each part-worth utility, 95% confidence intervals were manually calculated in Microsoft Excel 2013, in order to determine significant differences between the levels of each attribute (main effects). Part-worth utilities were interpreted relative to the least favorable level within each environmental attribute (enclosed street setting, no separation, 70 km/h, very uneven cycle path), and received a utility score equal to zero. To interpret the two-way interaction effects between street setting and micro-environmental attributes, total utilities of a street were calculated in Microsoft Excel 2013, summing the part-worth utilities from the different attributes. For example, the total utility of an even cycle path in an open street setting, with no separation from motorised traffic and a 70 km/h speed limit was calculated by summing the part-worth utility of these four environmental attributes. Furthermore, 95% confidence intervals were calculated for the average total utilities and were presented in graphs to clarify the results.

In addition to the utilities, average relative importance scores were calculated for both the main effects and the interaction effects. In the main effects, these values demonstrate the maximum effect each environmental attribute has upon street choice for the total sample [33]. The average importance of each environmental attribute was calculated as follows: the range of the highest and the lowest part-worth utility within one environmental attribute, divided by the sum of the ranges of the part-worth utilities of the four attributes. For the interaction effects, the same calculations were performed, but the ranges were calculated between the highest and the lowest value of the interaction effect of a particular micro-environmental attribute within one of the three street settings, relative to the sum of the three ranges from the micro-environmental attributes. To obtain an overview of the fit of each model, root likelihood (RLH) values were interpreted. RLH range from zero to one, with a higher value indicating a better fit of model. Statistical significance was set at *α* = 0.05.

## Results

### Participants

In total, 620 children (response rate = 88.2%) participated in the study. The children not participating in the study did not obtain consent from their parents to complete the questionnaire (6.7%) or were absent on the day of data collection in their school (5.1%). Of the 703 children, 317 parents (response rate = 45.1%) provided complete data. In the current analysis, children were only included if parental data were available to enable comparison between parents and children. This resulted in the inclusion of 305 children-parent pairs. Children whose parents participated in the study self-reported lower BMI-scores (16.8 kg/m^2^ vs. 17.3 kg/m^2^; t = 2.24; p = 0.026) and were less frequently the decision maker of transport mode to school compared to children whose parents did not complete the questionnaire (7.9% vs 17.0%; *χ*^2^ = 14.86, p = 0.005). No other differences were observed between children whose parents participated in the study and those who did not. Descriptive characteristics of the participating parents and children can be found in Table 1.

**Table 1:** **Descriptive characteristics of the sample (n = 305)**

**Characteristics of the children**	
Age (years; mean, SD)	11.3 (0.6)
Sex (% girls)	48.2
Grade (% 5th grade)	49.5
Independent mobility (% not allowed to cycle on its own)	16.3
Living area	
Urban (%)	44.7
Suburban (%)	46.4
Rural (%)	9.2
**Characteristics of the parents**	
Age (years; mean, SD)	41.9 (5.4)
Sex (% mothers)	78.7
Educational level	
Primary education or less (%)	2.6
Secondary education (%)	33.8
Tertiary education (%)	63.6
Marital status	
Married (%)	76.7
Divorced/widowed/never married (%)	12.1
Cohabiting (%)	11.1

### Choice tasks children

#### Main effects

For children, the most important attributes influencing their decision regarding the street in which they preferred to cycle included evenness of the cycle path (average importance = 32.3%; 95% CI = 29.1, 34.8) and the speed limit (29.2%; 95% CI = 27.1, 31.3), which did not differ significantly from each other. The street setting (20.8%; 95% CI = 19.1, 22.4) and degree of separation (17.7%; 95% CI = 16.4, 19.1) were significantly less important, compared to the evenness of the cycle path and the speed limit.

Within the environmental attributes, all part-worth utilities significantly differed from each other (p < 0.05). Children preferred to cycle in open street settings (average part-worth utility = 2.13; 95% CI = 1.88, 2.37) compared to a half open street setting (0.85; 95% CI = 0.71, 0.98), which was preferred over an enclosed street setting (the reference level). Additionally, the analysis found clear preferences for the anticipated most attractive level (i.e. even cycle path, separation with a hedge and 30 km/h speed limits) over the intermediate and the unattractive level of each environmental factor. The model including all main effects had a RLH of 0.82.

#### Interaction effects

**Figure 2:**
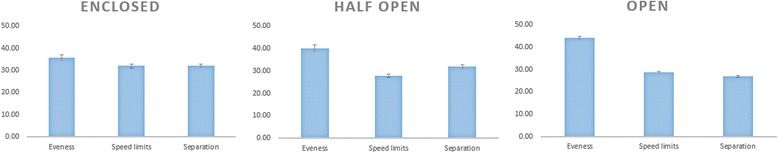
The relative importance of the micro-environmental attributes across the three street settings among children.

The relative importance of each micro-environmental attribute in each street setting is shown in Figure 2. In an enclosed street setting, no significant differences in importance between the three micro-environmental attributes was observed: evenness (35.8%; 95% CI = 33.1, 38.4), speed limit (32.1%; 95% CI = 30.4, 33.8) and degree of separation (32.1%; 95% CI = 30.9, 33.4) were equally important. In a half open street setting, the evenness of the cycle path (40.2%; 95% CI = 37.2, 43.2) was significantly more important than the degree of separation (32.0%; 95% CI = 30.3, 35.6) and speed limit (28.0%; 95% CI = 26.3, 29.4). In an open street setting, the evenness of the cycle path (44.2%; 95% CI = 42.7, 45.6) was significantly more important than the speed limit (28.8%; 95% CI = 28.0, 29.6) and degree of separation from motorised traffic (27.0%; 95% CI = 26.1, 27.9). An overview of all part-worth utilities for each effect can be found in Additional file 1.

##### Interaction between street setting and evenness of the cycle path

**Figure 3:**
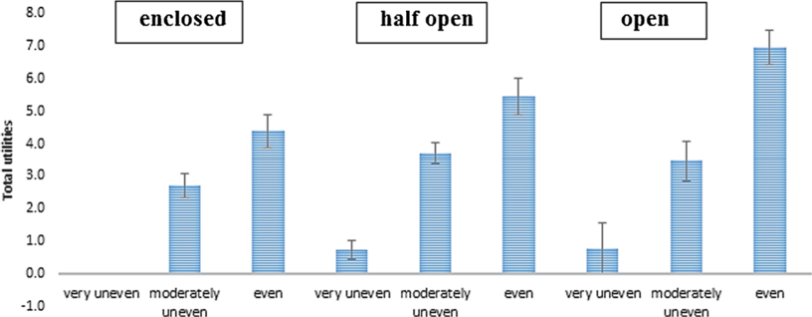
The effect of evenness of the cycle path across the different street settings among children (reference category = very uneven).

Adding the interaction term between the street setting and evenness of the cycle path increased the model fit including the main effects (RLH = 0.84). An even cycle path was preferred to a moderately and very uneven cycle path across all street settings (see Figure 3). However, the strength of the effect of an even (vs. very uneven) cycle path differed across the street settings. The effect of an even vs. very uneven cycle path was stronger in an open (difference in effect = 1.79; 95% CI = 1.04, 2.53) and half open (0.32; 95% CI = 0.18, 0.48) street setting compared to an enclosed street setting. The effect of a moderately uneven versus a very uneven cycle path did not significantly differ across the three street settings.

##### Interaction between street setting and speed limitation

**Figure 4:**
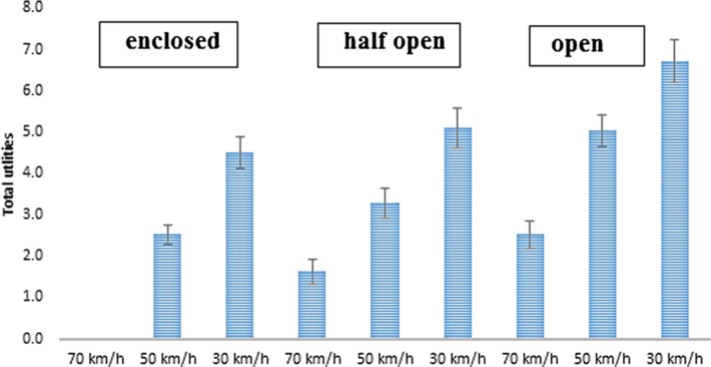
The effect of speed limitation across the different street settings among children (reference category = 70 km/h).

Adding the interaction term between street setting and speed limitation increased the fit of model including all main effects (RLH = 0.84). A speed limitation of 30 km/h was preferred across all street settings, although the magnitude of the effects differed across street settings (see Figure 4). The effect of a speed limitation of 30 km/h (vs. 70 km/h) was weaker in an open (−0.31; 95% CI = −0.41, −0.21) and a half open (−1.02; 95% CI = −1.18, −0.87) street setting compared to an enclosed street setting. The effect of a 50 km/h speed limitation (vs. 70 km/h) was similar between an enclosed and open street setting, but was significantly stronger than in a half open street setting (half open vs. open = −0.86; 95% CI = −0.97, −0.75; half open vs. enclosed = −0.86; 95% CI = −1.04; −0.68).

##### Interaction between street setting and degree of separation

**Figure 5:**
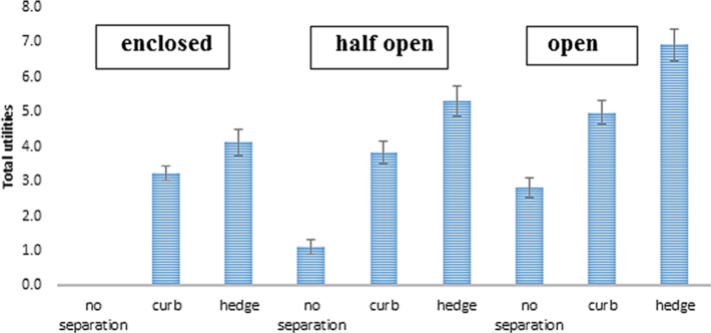
The effect of degree of separation across the different street settings among children (reference category = no separation).

Adding the interaction term between street setting and degree of separation increased the fit of model including all main effects (RLH = 0.84). Across all street settings, a hedge as a separation from motorised traffic was preferred to no separation between motorised traffic and the cycle path. The magnitude of this effect was the same across all street settings. The effect of a curb (vs. no separation) was stronger in an enclosed street setting compared to half open (−0.52; 95% CI = −0.72, −0.33) and open street setting (−1.05; 95% CI = −1.19, − 0.92) (see Figure 5).

### Choice tasks parents

#### Main effects

The speed limit in the street (average importance = 36.4%; 95% CI = 34.7, 38.1) and degree of separation with motorised traffic (35.1%; 95% CI = 33.5, 36.8) were of highest importance among parents and did not significantly differ from each other. Parents’ street choice was significantly less influenced by evenness of the cycle path (21.1%; 95% CI =19.2, 23.1) while the street setting was considered least important (7.3%; 95% CI = 6.6, 8.0).

No difference was observed in part-worth utilities between an open (average part-worth utility = 0.37; 95% CI = 0.21, 0.52) or a half open (0.44; 95% CI = 0.32, 0.56) street setting, but both street settings were preferred to an enclosed (reference level) street setting. Furthermore, parents preferred their child to cycle along streets depicting the anticipated most attractive levels of the other three environmental factors (i.e. even cycle path, separation with a hedge and 30 km/h speed limits) compared to the intermediate and unattractive level. The model including all main effects had a RLH of 0.88.

#### Interaction effects

**Figure 6:**
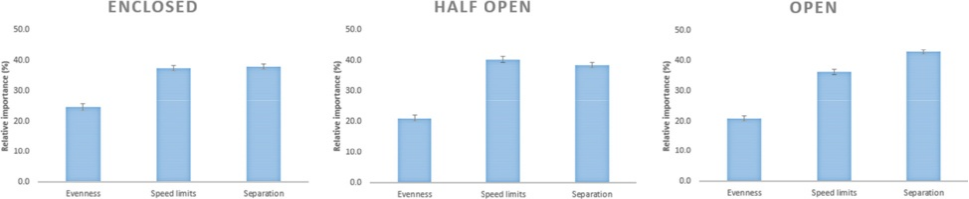
The relative importance of each micro-environmental attribute across the three different street settings among parents.

The relative importance of each micro-environmental attribute in each street setting is shown in Figure 6. In an enclosed street setting, degree of separation (37.9%; 95% CI = 36.2, 39.6) and speed limitation (37.4%; 95% CI = 35.7, 39.2) were the most important attributes regarding parents’ preferred street for their child to cycle along. Evenness of the cycle path was a less important attribute in an enclosed street setting (24.7%; 95% CI = 22.6, 26.8). In a half open street setting, the same relative importance scores were observed, i.e., degree of separation (38.5%; 95% CI = 36.7, 40.3) and speed limit (40.3%; 95% CI = 38.6, 42.1) were the most important attributes, followed by evenness of the cycle path (21.2%; 95% CI = 19.2, 23.1). In an open street setting, degree of separation (42.9%; 95% CI = 41.4, 44.5) was the most important attribute, which was significantly more important than speed limit (36.2; 95% CI = 34.4, 37.9) and evenness of the cycle path (20.9%; 95% CI = 19.0, 22.7).

##### Interaction effect of street setting and evenness of cycle path

**Figure 7:**
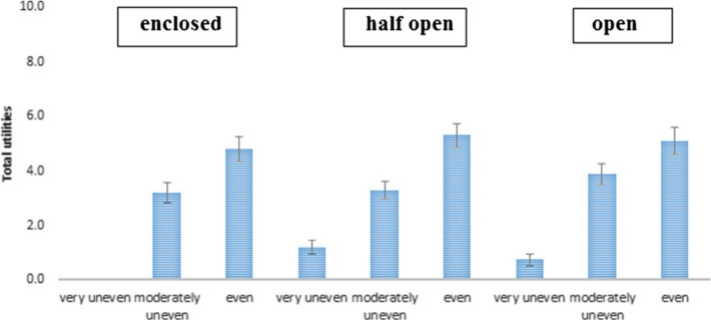
The effect of evenness of the cycle path across the different street settings among parents (reference category = very uneven).

Adding the interaction term between street setting and evenness of cycle path increased the fit of the model including all main effects (RLH = 0.89). An even cycle path was preferred to a moderately and very uneven cycle path across all street settings (see Figure 7). The effect of an even (vs. very uneven) cycle path was stronger in an enclosed street setting compared to a half open (−0.70; 95% CI = −0.86, −0.54) and open (−0.45; 95% CI = −0.55, −0.35) street setting. The effect of a moderately uneven (vs. very uneven) cycle path was also stronger in an enclosed street setting compared to a half open (−1.11; 95% CI = −1.34, −0.89) street, but not compared to an open street setting (−0.07; 95% CI = −0.18, 0.05).

##### Interaction effect of street setting and speed limit

**Figure 8:**
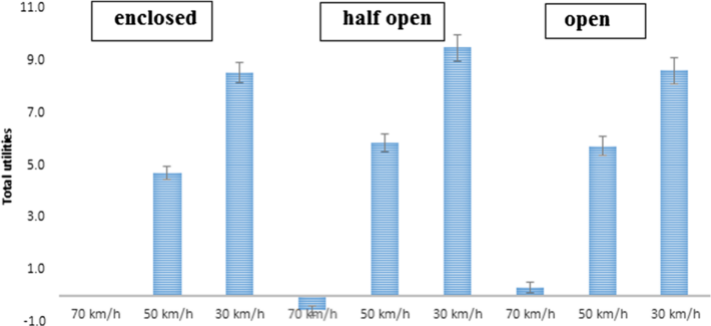
The effect of speed limits across the different street settings among parents (reference category = 70 km/h).

Adding the interaction term between street setting and speed limit increased the fit of the model including all main effects (RLH = 0.89). A speed limit of 30 km/h was preferred to 70 km/h and 50 km/h in all street settings. The effect of a speed limit of 30 km/h compared to 70 km/h was stronger in a half open compared to an enclosed (−1.50; 95% CI = −1.68, −1.33) and an open (−1.78; 95% CI = −1.93, −1.63) street setting (see Figure 8). The effect of a speed limit of 50 km/h compared to 70 km/h was stronger in an open street setting compared to an enclosed (−0.67; 95% CI = −0.78, −0.57) and a half open (−1.00; 95% CI = −1.11, −0.89) street setting.

##### Interaction effect of street setting and degree of separation

**Figure 9:**
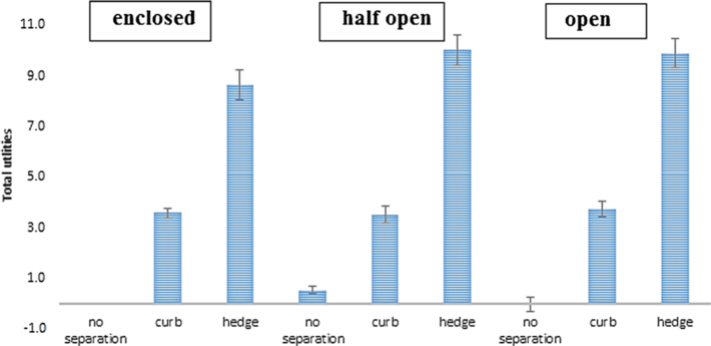
The effect of degree of separation across the different street settings among parents (reference category = no separation).

Adding the interaction term between street setting and degree of separation increased the fit of the model including all main effects (RLH = 0.90). Parents preferred a hedge as a way of separating the cycle path from motorised traffic across all street settings (see Figure 9). The effect of separation by a hedge compared to no separation was stronger in an open compared to half open (−0.44; 95% CI = −0.98, −0.68) and enclosed (−1.26; 95% CI = −1.43, −1.09) street setting. The effect of a curb versus no separation from motorised traffic was stronger in an open compared to half open (−0.77; 95 CI% = −0.94, −0.61) street setting, but did not significantly differ from the effect in an enclosed street setting (open vs. enclosed −0.18; 95% CI = −0.01, 0.37) street setting.

## Discussion

The aims of the current study were to identify whether micro-environmental factors influence the choice of routes for children to cycle along and whether the effects of these micro-environmental factors differ across street settings. Panoramic manipulated photographs integrated in CBC analysis were used to examine these innovative research questions.

The present study found that each level within the environmental attributes significantly differed from each other, indicating they had an influence on street preference for children’s cycling for transport. This was reflected in the main effects, where the most attractive level within each factor (i.e. even cycle path, 30 km/h speed restrictions and a separation between motorised traffic and the cycle path with a hedge) was preferred by both the children and their parents. Furthermore, children preferred to cycle in open street settings, while parents preferred both open and half open street settings. Real-life improvements in each of the three micro-environmental attributes may well influence the supportiveness of a street for transportation cycling. Furthermore, the main effects highlighted that among parents, traffic speed and degree of separation were the most important factors to prefer a street to let their child cycle along. This implies that motorised traffic speed regulations (or design to reduce motorised traffic speed) offer a possible strategy to increase the appeal of children’s cycling routes. This is consistent with several reviews on children’s active transport showing consistent negative cross-sectional associations between traffic speed and children’s active transport [9,21,22].

Furthermore, children and their parents preferred the street with a cycle path separated from the road by a hedge, rather than a curb or no separation. For parents, a curb was considered as good an alternative as a hedge for separating their children from passing motorised traffic during a cycle trip. In contrast, children’s lower preference for a curb may be due to perceived difficulties in accessing the cycle track over the curb. The preference for a separation is consistent with findings of studies among adults which concluded that having any separation from motorised traffic might be associated with an increase in perceived safety for cyclists [39]. Our findings are also consistent with previous studies among children [9,40] in which parental safety concerns, assessed in the current study as speed limit and degree of separation from motorised traffic, played an important role in their street preference. Safety should therefore be considered as the key priority for strategies to increase children’s cycling.

There were some other differences in findings between children and parents. No parental preference was observed for an open or a half open street setting. Some parents may prefer a higher residential density to let their child cycle, because of social control and stranger danger [41], while other parents may like the openness of a street for cycling which resulted in no clear preference between both street settings. In contrast, children preferred to cycle in open street settings, which may be explained by the preference of natural elements over building environments [42] and thus preference for aesthetic features rather than safety issues. Additionally, it was noteworthy that children’s choices focused more on the evenness of the cycle path across all different street settings, while their parents’ choices focused more on the separation between the cycle path and traffic. Evenness of the cycle path may be important for children as an uneven cycling path may hamper cycling, making cycling less pleasant. Additionally, previous studies also indicated that children focus more on evenness compared to their parents as children experience this difficulties more extensively [43,44]. Furthermore, it has been shown that bad cycle path conditions distract cyclists from motorised traffic, which may cause unsafe traffic situations [30]. Cycling on an even road surface offers more comfort for cyclists, and previous research has shown that an uneven surface was an important cause (18%) of bicycle accidents [45]. As children are less experienced and have lower cycling skills compared to adolescents or adults, cycling on uneven surfaces may cause an additional difficulty for them to cycle safely. So in addition to create new cycle infrastructure, communities should make sure to keep cycle paths well maintained to keep the surface even for the children. Parents’ focus on the separation of the cycle track with motorised traffic reflects the great importance they attach to traffic safety for their children [18]. Given the differences between choices of the children and their parents and the likely influence of each on the child’s transport behavior, future studies should consider both perspectives.

The observed significant interaction effects implied that the difference in effect between, for example, an even and a very uneven cycle path, was stronger in one street setting compared to other street settings. For example, among children, the difference between an even and a very uneven cycle path was strongest in an open street setting, compared to a half-open or enclosed street setting.

The significant interaction effects were also reflected in the relative importance of each micro-environmental factor across the street settings. For example, among parents, degree of separation from the road and speed limit were equally important in an enclosed and half open street setting, but degree of separation was the most important factor in an open street setting. This may suggest that in open streets with a lower building density, a separation between the street and the cycle path may be somewhat more effective when aiming to improve the appeal of the environment for transportation cycling. However, it should be noted that these interaction effects were very small, which limits its practical application in indicating which environmental factor should receive highest priority in each street setting. Additionally, adding interaction effects to the four main effects only resulted in small increases in the fit of the model (maximum increase of RLH was 0.02 in both models). These findings rather indicate that changing one or more micro-environmental factors in a street is an effective strategy to increase the appeal of a street for children’s transportation cycling, irrespective of the characteristics of the street setting. As changing micro-environmental factors in existing environments is more feasible and less expensive than substantial changes to more robust macro-environmental factors such as street connectivity, residential density or distances to destinations, these results are very informative for urban planners, researchers and other practitioners who are planning to conduct structural changes in the physical environment. This information should be integrated in the development of on-site experimental research, where the effect of changing micro-scale environmental factors can be examined on children’s transportation behavior.

This study was the first to use CBC analysis to examine physical environmental factors influencing street preference for transportation cycling among children. This method has mainly been used in marketing research to establish the relative importance of different characteristics of a product [33], and has now successfully been used to consider the effect of different environmental attributes in a hypothetical street setting. Additionally, it was previously unknown whether micro-environmental attributes could influence the preferred street for children’s cycling. Whether the effect of each micro-environmental attribute was similar across different street settings was also unknown. We consider this laboratory study using manipulated photographs useful before conducting on-site experimental research, as it represents a practical explorative step to get insight in which environmental factors may have an effect on the supportiveness of real streets. Before doing a time-consuming and costly change to a street, it makes sense to test likely effects through a simulation like the present study.

The experimental use of manipulated photographs allowed the study of potentially causal relationships between several environmental attributes and preference for these attributes for cycling for transport among children, rather than focussing on one environmental attribute separately. Studying multiple environmental factors at the same time also mimics real life situations, where combinations of factors influence the decision of where to cycle. Furthermore, a reasonably large sample of both children and parents completed the questionnaire, allowing comparisons between children and their parents. A final strength of the study was the use of conjoint analysis, which allowed studying the relative importance of each environmental attribute without showing participants all possible combinations of factors, which limited the time needed to complete the total questionnaire to a maximum of 30 minutes for parents and 20 minutes for children.

Despite these strengths, there are a few limitations of the current study that need to be acknowledged. A main limitation was the inclusion of only four environmental attributes manipulated in the photographs. Therefore, the main effects must be interpreted with caution, as not all possible environmental factors influencing the choice for a specific street were included [33]. The four included attributes were previously found to be related to cycling for transport among children in cross-sectional studies, and may therefore have had a substantial influence on the choices [18,28,29]. The results of conjoint analysis depend on the number of attributes included in the study and combinations of the components. Therefore, future studies using conjoint methodology should examine other possible influencing factors for the specific research question. In the current analysis, we included only children for whom parental data were available. We also performed the analysis for the total sample of children and observed no differences with the results presented here. Additionally, more than half of the parents indicated to have obtained a tertiary degree, which is much higher than the prevalence (32.5%) in the Flemish adult population [46]. Future research could test how well the present results apply to children and parents of lower socio-economic or educational status.

Despite the benefits of using manipulated photographs, this study did not test the actual cycling behaviour of the children. Future studies could observe whether streets that have the desired attributes attract more children cyclists. Additionally, studying the causal relation between changing the physical environment and children’s cycling behaviour is essential to get insight on how the physical environment affects children’s transportation cycling.

## Conclusions

For children, evenness of the cycle path and the speed limit of the street were the most important attributes, while for parents it was the speed limit and degree of separation from motorised traffic. Although most interactions between the different street settings and the micro-environmental attributes were statistically significant, the effects of the micro-environmental attributes were very similar across the different street settings with only small differences in magnitude of the effects according to street setting. Thus interventions aiming to improve the built environment in order to increase cycling for transport may benefit from changes in micro-environmental factors, which are easier to conduct and less expensive than changing macro-environmental factors. Future research in real-life settings are needed to determine whether the current findings can be linked with children’s actual cycling behaviour.

## Additional file

Additional file 1:
**All part-worth utilities for the environmental factors: the main effects and interaction effects.**

## References

[1] Telford RM, Telford RD, Cunningham RB, Cochrane T, Davey R, Waddington G. Longitudinal patterns of physical activity in children aged 8 to 12 years: the LOOK study. Int J Behav Nutr Phys Act. 2013;10. 10.1186/1479-5868-10-81 PMC3691664 24456743

[2] Sallis JF (1993). Epidemiology of physical activity and fitness in children and adolescents. Crit Rev Food Sci Nutr.

[3] Trapp G, Giles-Corti B, Christian H, Bulsara M, Timperio A, McCormack G (2011). On your bike! a cross-sectional study of the individual, social and environmental correlates of cycling to school. Int J Behav Nutr Phys Act.

[4] Andersen LB, Wedderkopp N, Kristensen P, Moller NC, Froberg K, Cooper AR (2011). Cycling to school and cardiovascular risk factors: a longitudinal study. J Phys Act Health.

[5] Ostergaard L, Kolle E, Steene-Johannessen J, Anderssen S, Andersen L (2013). Cross sectional analysis of the association between mode of school transportation and physical fitness in children and adolescents. Int J Behav Nutr Phys Act.

[6] Carver A, Watson B, Shaw B, Hillman M (2013). A comparison study of children’s independent mobility in England and Australia. Children’s Geographies.

[7] Hillman M, Adams J, Whitelegg J (1990). One false move.

[8] Yeung J, Wearing S, Hills AP (2008). Child transport practices and perceived barriers in active commuting to school. Transport Res Pol Pract.

[9] Panter J, Jones A, van Sluijs E. Environmental determinants of active travel in youth: A review and framework for future research. Int J Behav Nutr Phys Act. 2008. 10.1186/1479-5868-5-34 PMC2483993 18573196

[10] Brug J, van Stralen MM, te Velde SJ, Chinapaw MJM, De Bourdeaudhuij I, Lien N (2012). Differences in weight status and energy-balance related behaviors among schoolchildren across Europe: The ENERGY-Project. PLoS One.

[11] Mobiel Vlaanderen. Rapporten Onderzoek Verplaatsingsgedrag Vlaanderen 4.4 (September 2011-September 2012) Vlaamse Overheid 2013.

[12] Baranowski T, Anderson C, Carmack C (1998). Mediating variable framework in physical activity interventions. How are we doing? How might we do better?. Am J Prev Med.

[13] Sallis JF, Cervero RB, Ascher W, Henderson KA, Kraft MK, Kerr J (2006). An ecological approach to creating active living communities. Annu Rev Public Health.

[14] Brockett C (1976). Toward a clarification of need hierarchy theory - some extensions of Maslows Conceptualization. Interpers Dev.

[15] Alfonzo MA (2005). To walk or not to walk? The hierarchy of walking needs. Environ Behav.

[16] D’Haese S, De Meester F, De Bourdeaudhuij I, Deforche B, Cardon G (2011). Criterion distances and environmental correlates of active commuting to school in children. Int J Behav Nutr Phys Act.

[17] Frank LD, Sallis JF, Saelens BE, Leary L, Cain K, Conway TL, et al. The Development of a Walkability Index: Application To the Neighborhood Quality of Life Study. Br J Sports Med 2009. 10.1136/bjsm.2009.058701 19406732

[18] Ghekiere A, Van Cauwenberg J, de Geus B, Clarys P, Cardon G, Salmon J, et al. Critical environmental factors of transportation cycling in children: a qualitative study using bike-along interviews. Plos One. 2014;9. http://journals.plos.org/plosone/article?id=10.1371/journal.pone.0106696 10.1371/journal.pone.0106696 PMC4175075 25250738

[19] De Meester F, Van Dyck D, De Bourdeaudhuij I, Deforche B, Sallis J, Cardon G (2012). Active living neighborhoods: is neighborhood walkability a key element for Belgian adolescents?. BMC Public Health.

[20] Leslie E, Kremer P, Toumbourou JW, Williams JW (2010). Gender differences in personal, social and environmental influences on active travel to and from school for Australian adolescents. J Sci Med Sport.

[21] Davison KK, Lawson CT (2006). Do attributes in the physical environment influence children’s physical activity? A review of the literature. Int J Behav Nutr Phys Activ.

[22] Ding D, Sallis JF, Kerr J, Lee S, Rosenberg DE (2011). Neighborhood environment and physical activity among youth: a review. Am J Prev Med.

[23] Kusenbach M (2003). Street phenomenology: the go-along as ethnographic research tool. Ethnography.

[24] Mitra R, Buliung RN (2012). Built environment correlates of active school transportation: neighborhood and the modifiable areal unit problem. J Transport Geogr.

[25] Chaix B, Meline J, Duncan S, Merrien C, Karusisi N, Perchoux C (2013). GPS tracking in neighborhood and health studies: a step forward for environmental exposure assessment, a step backward for causal inference?. Health Place.

[26] Van Cauwenberg J, Van Holle V, De Bourdeaudhuij I, Clarys P, Nasar J, Salmon J (2014). Using manipulated photographs to identify features of streetscapes that may encourage older adults to walk for transport. PloS One.

[27] Mertens L, Van Holle V, De Bourdeaudhuij I, Deforche B, Salmon J, Nasar J (2014). The effect of changing micro-scale physical environmental factors on an environment’s invitingness for transportation cycling in adults: an exploratory study using manipulated photographs. Int J Behav Nutr Phys Act.

[28] Fraser SD, Lock K (2011). Cycling for transport and public health: a systematic review of the effect of the environment on cycling. Eur J Publ Health.

[29] Carver A, Timperio A, Crawford D (2008). Playing it safe: the influence of neighbourhood safety on children’s physical activity—A review. Health Place.

[30] Vansteenkiste P, Zeuwts L, Cardon G, Philippaerts R, Lenoir M (2014). The implications of low quality bicycle paths on gaze behavior of cyclists: A field test. Transp Res Pt F-Traffic Psychol Behav.

[31] Ayachi FS, Dorey J, Guastavino C (2015). Identifying factors of bicycle comfort: an online survey with enthusiast cyclists. Appl Ergon.

[32] Nasar JL (2008). Assessing perceptions of environments for active living. Am J Prev Med.

[33] Orme BK (2006). Getting started with Conjoint Analysis: Strategies for Product Design and Pricing Research.

[34] Nordh H, Hartig T, Hagerhall CM, Fry G (2009). Components of small urban parks that predict the possibility for restoration. Urban Forestry and Urban Greening.

[35] Winters M, Friesen MC, Koehoorn M, Teschke K (2007). Utilitarian bicycling: a multilevel analysis of climate and personal influences. Am J Prev Med.

[36] Craig CL, Marshall AL, Sjostrom M, Bauman AE, Booth ML, Ainsworth BE (2003). International physical activity questionnaire: 12-country reliability and validity. Med Sci Sports Exerc.

[37] Johnson RaOB. Getting the most from CBC, Technical paper available from the World Wide Web. In Book Getting the most from CBC, Technical paper available from the World Wide Web (Editor ed.^eds.). City; 2003.

[38] Allenby GM, Arora N, Ginter JL. On the heterogeneity of demand. J Mark Res. 1998;384–389.

[39] Dill J, McNeil N. Four Types of Cyclists? Examination of Typology for Better Understanding of Bicycling Behavior and Potential. Transp Res Rec. 2013;129–138.

[40] Davison KK, Werder JL, Lawson CT (2008). Children’s active commuting to school: current knowledge and future directions. Prev Chronic Dis.

[41] Foster S, Villanueva K, Wood L, Christian H, Giles-Corti B (2014). The impact of parents’ fear of strangers and perceptions of informal social control on children’s independent mobility. Health Place.

[42] Kaplan S (1995). The restorative benefits of nature: toward an integrative framework. J Environ Psychol.

[43] Lukashok AK, Lynch K (1956). Some childhood memories of the city. J Am Inst Plann.

[44] Lynch K (1959). A walk around the block.

[45] Nyberg P, Bjornstig U, Bygren LO (1996). Road characteristics and bicycle accidents. Scand J Soc Med.

[46] Flemish Employment and Vocational Training. Low skilled people in the Flemish labor market. In Book Low skilled people in the Flemish labor market (Editor ed.^eds.). City; 2010.

