# Supplementary material for: Assessing cycling-friendly environments for children: are micro-environmental factors equally important across different street settings?
Source: Int J Behav Nutr Phys Act. 2015 May 2;12:54. doi: 10.1186/s12966-015-0216-2 (PMC4436842; doi:10.1186/s12966-015-0216-2)
Supplement: Additional file 1: — All part-worth utilities for the environmental factors: the main effects and interaction effects. [file 12966_2015_216_MOESM1_ESM.docx]

Additionial file

# Main effects

## Children

Additional Table 1. Main effects of the environmental factors among children, expressed in part-worth utilities

| Environmental factor | Part-worth utility | Standard Error | Lower 95% CI | Upper 95% CI |
| --- | --- | --- | --- | --- |
| enclosed (ref cat) |  |  |  |  |
| half-open | 0.85 | 0.07 | 0.71 | 0.98 |
| open | 2.13 | 0.12 | 1.88 | 2.37 |
| very uneven (ref cat) |  |  |  |  |
| moderately uneven | 2.65 | 0.11 | 2.43 | 2.86 |
| even | 4.18 | 0.22 | 3.75 | 4.61 |
| 70 km/h (ref cat) |  |  |  |  |
| 50 km/h | 1.93 | 0.09 | 1.76 | 2.10 |
| 30 km/h | 3.64 | 0.16 | 3.33 | 3.95 |
| no separation (ref cat) |  |  |  |  |
| separation by a curb | 0.37 | 0.09 | 0.20 | 0.54 |
| separation by a hedge | 1.87 | 0.11 | 1.65 | 2.09 |

## Parents

Additional Table 2. Main effects of the environmental factors among parents, expressed in part-worth utilities

| Environmental factor | Part-worth utility | Standard Error | Lower 95% CI | Upper 95% CI |
| --- | --- | --- | --- | --- |
| enclosed (ref cat) |  |  |  |  |
| half-open | 0.44 | 0.06 | 0.32 | 0.56 |
| open | 0.37 | 0.08 | 0.21 | 0.52 |
| very uneven (ref cat) |  |  |  |  |
| moderately uneven | 2.62 | 0.07 | 2.48 | 2.76 |
| even | 4.05 | 0.22 | 3.61 | 4.49 |
| 70 km/h (ref cat) |  |  |  |  |
| 50 km/h | 4.86 | 0.10 | 4.67 | 5.06 |
| 30 km/h | 7.99 | 0.20 | 7.60 | 8.38 |
| no separation (ref cat) |  |  |  |  |
| separation by a curb | 2.81 | 0.17 | 2.48 | 3.14 |
| separation by a hedge | 7.78 | 0.22 | 7.34 | 8.21 |

# Interaction effects

## Children

| Additional Table 3. Interaction between evenness of the cycle path and street setting among children | | | | |
| --- | --- | --- | --- | --- |
| Environmental factor | part-worth utility | Standard Error | Lower 95% CI | Upper 95% CI |
| E * very uneven (ref cat) |  |  |  |  |
| E * moderately uneven | 2.71 | 0.19 | 2.34 | 3.08 |
| E * even | 4.40 | 0.26 | 3.89 | 4.90 |
| HO * very uneven | 0.74 | 0.15 | 0.44 | 1.03 |
| HO * moderately uneven | 3.71 | 0.16 | 3.40 | 4.03 |
| HO * even | 5.45 | 0.29 | 4.88 | 6.02 |
| O * very uneven | 0.77 | 0.40 | -0.01 | 1.56 |
| O * moderately uneven | 3.46 | 0.32 | 2.84 | 4.09 |
| O * even | 6.96 | 0.26 | 6.44 | 7.47 |
| E= enclosed; HO = half-open; O= open | |  |  |  |

| Additional Table 4. Interaction between speed limitation and street setting among children | | | | |
| --- | --- | --- | --- | --- |
| Environmental factor | part-worth utility | Standard Error | Lower 95% CI | Upper 95% CI |
| E* 70 km/h (ref cat) |  |  |  |  |
| E* 50 km/h | 2.51 | 0.12 | 2.28 | 2.74 |
| E * 30 km/h | 4.51 | 0.19 | 4.13 | 4.89 |
| HO * 70 km/h | 1.62 | 0.16 | 1.31 | 1.93 |
| HO * 50 km/h | 3.27 | 0.18 | 2.92 | 3.63 |
| HO * 30 km/h | 5.10 | 0.24 | 4.64 | 5.57 |
| O * 70 km/h | 2.51 | 0.17 | 2.17 | 2.86 |
| O *50 km/h | 5.03 | 0.20 | 4.64 | 5.41 |
| O * 30 km/h | 6.72 | 0.26 | 6.20 | 7.23 |
| E= enclosed; HO = half-open; O= open | |  |  |  |

| Additional Table 5. Interaction between degree of separation and street setting among children | | | | | | |
| --- | --- | --- | --- | --- | --- | --- |
| Environmental factor | part-worth utility | | Standard Error | | Lower 95% CI | Upper 95% CI |
| E * no separation (ref cat) |  | |  | |  |  |
| E * curb separation | 3.23 | | 0.11 | | 3.03 | 3.44 |
| E * hedge separation | 4.12 | | 0.19 | | 3.74 | 4.49 |
| HO * no separation | 1.11 | | 0.11 | | 0.89 | 1.32 |
| HO * curb separation | 3.82 | | 0.17 | | 3.49 | 4.15 |
| HO * hedge separation | 5.31 | | 0.21 | | 4.89 | 5.73 |
| O * no separation | 2.80 | | 0.14 | | 2.52 | 3.08 |
| O * curb separation | 4.98 | | 0.17 | | 4.64 | 5.32 |
| O * hedge separation | 6.92 | | 0.23 | | 6.46 | 7.37 |
| E= enclosed; HO = half-open; O= open | | |  | |  |  |
| Additional Table 6. Relative importance of the environmental factors among children | | | | | | |
| Environmental factor | | Relative importance (%) | | Standard Error | Lower 95% CI | Upper 95% CI |
| Street setting | | 20.8 | | 0.84 | 19.15 | 22.45 |
| Evenness of the cycle path | | 32.3 | | 1.29 | 29.77 | 34.83 |
| Speed limitation | | 29.2 | | 1.07 | 27.10 | 31.30 |
| Degree of separation | | 17.7 | | 0.7 | 16.33 | 19.07 |
|  | |  | |  |  |  |
| *Within enclosed street setting* | |  | |  |  |  |
| Evenness of the cycle path | | 35.77 | | 1.35 | 33.13 | 38.42 |
| Speed limitation | | 32.1 | | 0.88 | 30.37 | 33.83 |
| Degree of separation | | 32.13 | | 0.65 | 30.86 | 33.4 |
|  | |  | |  |  |  |
| *Within half-open street setting* | |  | |  |  |  |
| Evenness of the cycle path | | 40.17 | | 1.52 | 37.19 | 43.16 |
| Speed limitation | | 27.86 | | 0.80 | 26.29 | 29.43 |
| Degree of separation | | 31.96 | | 0.83 | 30.34 | 33.59 |
|  | |  | |  |  |  |
| *Within open street setting* | |  | |  |  |  |
| Evenness of the cycle path | | 44.15 | | 0.72 | 42.74 | 45.56 |
| Speed limitation | | 28.81 | | 0.39 | 28.04 | 29.58 |
| Degree of separation | | 27.04 | | 0.46 | 26.13 | 27.94 |

## Parents

| Additional Table 7. Interaction between evenness of the cycle path and street setting among parents | | | | |
| --- | --- | --- | --- | --- |
| Environmental factor | Part-worth utility | Standard Error | Lower 95% CI | Upper 95% CI |
| E * very uneven (ref cat) |  |  |  |  |
| E * moderately uneven | 3.21 | 0.09 | 3.04 | 3.39 |
| E * even | 4.81 | 0.08 | 4.66 | 4.97 |
| HO * very uneven | 1.19 | 0.28 | 0.64 | 1.74 |
| HO * moderately uneven | 3.29 | 0.10 | 3.09 | 3.48 |
| HO * even | 5.30 | 0.05 | 5.20 | 5.40 |
| O * very uneven | 0.73 | 0.23 | 0.28 | 1.19 |
| O * moderately uneven | 3.88 | 0.07 | 3.74 | 4.02 |
| O * even | 5.10 | 0.25 | 4.62 | 5.58 |
| E= enclosed; HO = half-open; O= open | |  |  |  |

| Additional Table 8. Interaction between speed limitation and street setting among parents | | | | | | | |
| --- | --- | --- | --- | --- | --- | --- | --- |
| Environmental factor | Part-worth utility | | | Standard Error | | Lower 95% CI | Upper 95% CI |
| E* 70 km/h (ref cat) |  | | |  | |  |  |
| E* 50 km/h | 4.70 | | | 0.14 | | 4.43 | 4.97 |
| E * 30 km/h | 8.53 | | | 0.20 | | 8.13 | 8.94 |
| HO * 70 km/h | -0.54 | | | 0.09 | | -0.72 | -0.36 |
| HO * 50 km/h | 5.84 | | | 0.17 | | 5.49 | 6.18 |
| HO * 30 km/h | 9.50 | | | 0.28 | | 8.95 | 10.05 |
| O * 70 km/h | 0.35 | | | 0.10 | | 0.15 | 0.54 |
| O *50 km/h | 5.72 | | | 0.19 | | 5.36 | 6.09 |
| O * 30 km/h | 8.60 | | | 0.26 | | 8.09 | 9.11 |
| E= enclosed; HO = half-open; O= open | | | |  | |  |  |
| Additional Table 9. Interaction degree of separation with street setting among parents | | | | | | | |
| Environmental factor | | | Part-worth utility | | Standard Error | Lower 95% CI | Upper 95% CI |
| E * no separation (ref cat) | | |  | |  |  |  |
| E * curb separation | | | 3.59 | | 0.22 | 3.16 | 4.02 |
| E * hedge separation | | | 8.68 | | 0.11 | 8.46 | 8.89 |
| HO * no separation | | | 0.53 | | 0.24 | 0.05 | 1.01 |
| HO * curb separation | | | 3.53 | | 0.19 | 3.15 | 3.91 |
| HO * hedge separation | | | 10.03 | | 0.09 | 9.86 | 10.21 |
| O * no separation | | | -0.03 | | 0.23 | -0.48 | 0.42 |
| O * curb separation | | | 3.74 | | 0.18 | 3.40 | 4.09 |
| O * hedge separation | | | 9.91 | | 0.28 | 9.37 | 10.46 |
| E= enclosed; HO = half-open; O= open | | | | |  |  |  |
| Additional Table 10. Relative importance of the environmental factors among parents | | | | | | | |
| Environmental factor | | Relative importance (%) | | | Standard Error | Lower 95% CI | Upper 95% CI |
| Street setting | | 7.3 | | | 0.36 | 6.59 | 8.01 |
| Evenness of the cycle path | | 21.1 | | | 1 | 19.14 | 23.06 |
| Speed limitation | | 36.4 | | | 0.36 | 35.69 | 37.11 |
| Degree of separation | | 35.1 | | | 0.84 | 33.45 | 36.75 |
|  | |  | | |  |  |  |
| *Within enclosed street setting* | |  | | |  |  |  |
| Evenness of the cycle path | | 24.68 | | | 1.06 | 22.61 | 26.75 |
| Speed limitation | | 37.45 | | | 0.88 | 35.73 | 39.16 |
| Degree of separation | | 37.87 | | | 0.88 | 36.15 | 39.59 |
|  | |  | | |  |  |  |
| *Within half-open street setting* | |  | | |  |  |  |
| Evenness of the cycle path | | 21.17 | | | 1.01 | 19.19 | 23.14 |
| Speed limitation | | 40.32 | | | 0.88 | 38.59 | 42.05 |
| Degree of separation | | 38.52 | | | 0.91 | 36.73 | 40.31 |
|  | |  | | |  |  |  |
| *Within open street setting* | |  | | |  |  |  |
| Evenness of the cycle path | | 20.88 | | | 0.96 | 18.99 | 22.76 |
| Speed limitation | | 36.18 | | | 0.89 | 34.44 | 37.92 |
| Degree of separation | | 42.94 | | | 0.81 | 41.36 | 44.53 |
